# Long-Term Outcomes of Prolonged Atlantoaxial Rotatory Fixation Managed Without Halo Vest Immobilization in a Child

**DOI:** 10.7759/cureus.114324

**Published:** 2026-08-11

**Authors:** Ryota Ito, Yusuke Oshita

**Affiliations:** 1 Orthopedic Surgery, SHOWA Medical University Northern Yokohama Hospital, Yokohama, JPN

**Keywords:** atlantoaxial joint, atlantoaxial rotatory fixation (aarf), child, remodeling therapy, torticollis

## Abstract

Atlantoaxial rotatory fixation (AARF) in children usually responds to conservative treatment, including rest and cervical collar immobilization. However, some patients experience persistent or recurrent deformity and require prolonged immobilization or more intensive treatment. Long-term outcomes of prolonged AARF managed without halo vest immobilization have rarely been reported. We report the case of an eight-year-old boy who presented with acute neck pain and torticollis without an identifiable trigger. Initial treatment with a cervical collar at a local clinic failed to improve his symptoms, and he was referred to our hospital two weeks after symptom onset. He was admitted and treated with 1.5-kg Glisson traction. Although his symptoms and cervical alignment initially improved, neck pain and torticollis repeatedly recurred after attempts to discontinue traction and transition to a cervical collar. Traction was therefore resumed several times. On hospital day 35, immobilization was changed to a cervicothoracic orthosis to provide better rotational control. Progressive sitting, standing, and gait training were subsequently introduced, and the patient was discharged home on hospital day 78. The cervicothoracic orthosis was changed to a cervical collar on day 238, and the collar was discontinued on day 315. Follow-up CT performed at six months showed improvement in cervical alignment and facet morphology. At the five-year follow-up, the patient remained asymptomatic and had a full cervical range of motion. CT demonstrated slight residual facet deformity without progression, bony ankylosis, or degenerative changes. This case demonstrates a favorable long-term clinical outcome after prolonged AARF managed with traction and external orthoses without general anesthesia, manipulation, or halo vest immobilization. In carefully selected pediatric patients in whom corrected alignment can be achieved and maintained, prolonged nonoperative management may be a treatment option. However, the contributions of natural growth and the natural history of AARF cannot be excluded, and further studies are required to determine the appropriate indications and efficacy of this approach.

## Introduction

Atlantoaxial rotatory fixation (AARF) is characterized by persistent pathological rotation of the atlas relative to the axis and typically presents with painful torticollis, a cock-robin posture, and restricted cervical motion. It predominantly affects children and adolescents. A nationwide survey using insurance claims data showed that 56.5% of patients were male, the peak age at onset was six years in both sexes, and recurrent AARF occurred in 6.2% of patients [1]. Trauma and infection are considered the most common precipitating factors, although AARF may also occur in association with inflammatory conditions, including Kawasaki disease [2,3].

Although most cases of pediatric torticollis are caused by benign conditions such as muscle spasm, infection, trauma, or AARF, intracranial or intraspinal disorders should also be considered. In a large emergency department cohort of 1,409 children, oncologic causes, including posterior fossa and brainstem tumors, accounted for 1.2% of torticollis presentations [4]. Therefore, further evaluation is important when torticollis is atypical or does not improve with initial treatment.

Most acute cases of AARF respond well to conservative treatment, including medication, cervical traction, closed manipulation, and subsequent orthotic immobilization [5]. However, delayed diagnosis, prolonged fixation, or recurrence after reduction may make reduction and maintenance of corrected alignment difficult. Persistent subluxation may lead to deformity of the superior facet of the axis and lateral inclination of the atlas, thereby increasing the risk of recurrence after reduction [5-7].

Remodeling therapy has been described for chronic or recurrent AARF and consists of closed reduction under general anesthesia followed by halo vest immobilization until improvement in the C2 facet deformity is confirmed [5-7]. This approach may help maintain reduction and prevent recurrent subluxation while avoiding surgical fixation. However, previously reported remodeling therapy has generally required halo vest immobilization. We report the case of an eight-year-old boy with prolonged AARF who was managed without manipulation under general anesthesia or halo vest immobilization. Neutral cervical alignment was maintained using traction followed by a cervicothoracic orthosis, and a favorable clinical outcome was maintained at the five-year follow-up despite slight residual facet deformity.

## Case presentation

An eight-year-old Japanese boy (height, 120 cm; weight, 24.1 kg) awoke with sudden neck pain and restricted cervical motion associated with torticollis. He had no preceding trauma or history of infection. Initial evaluations at two local orthopedic clinics led to the initiation of cervical collar immobilization; however, his symptoms did not improve, prompting referral to our hospital two weeks after symptom onset. He had no relevant past medical history and no family history of musculoskeletal or congenital disorders. There were no preceding upper respiratory tract infections or other prodromal symptoms.

**Figure 1:**
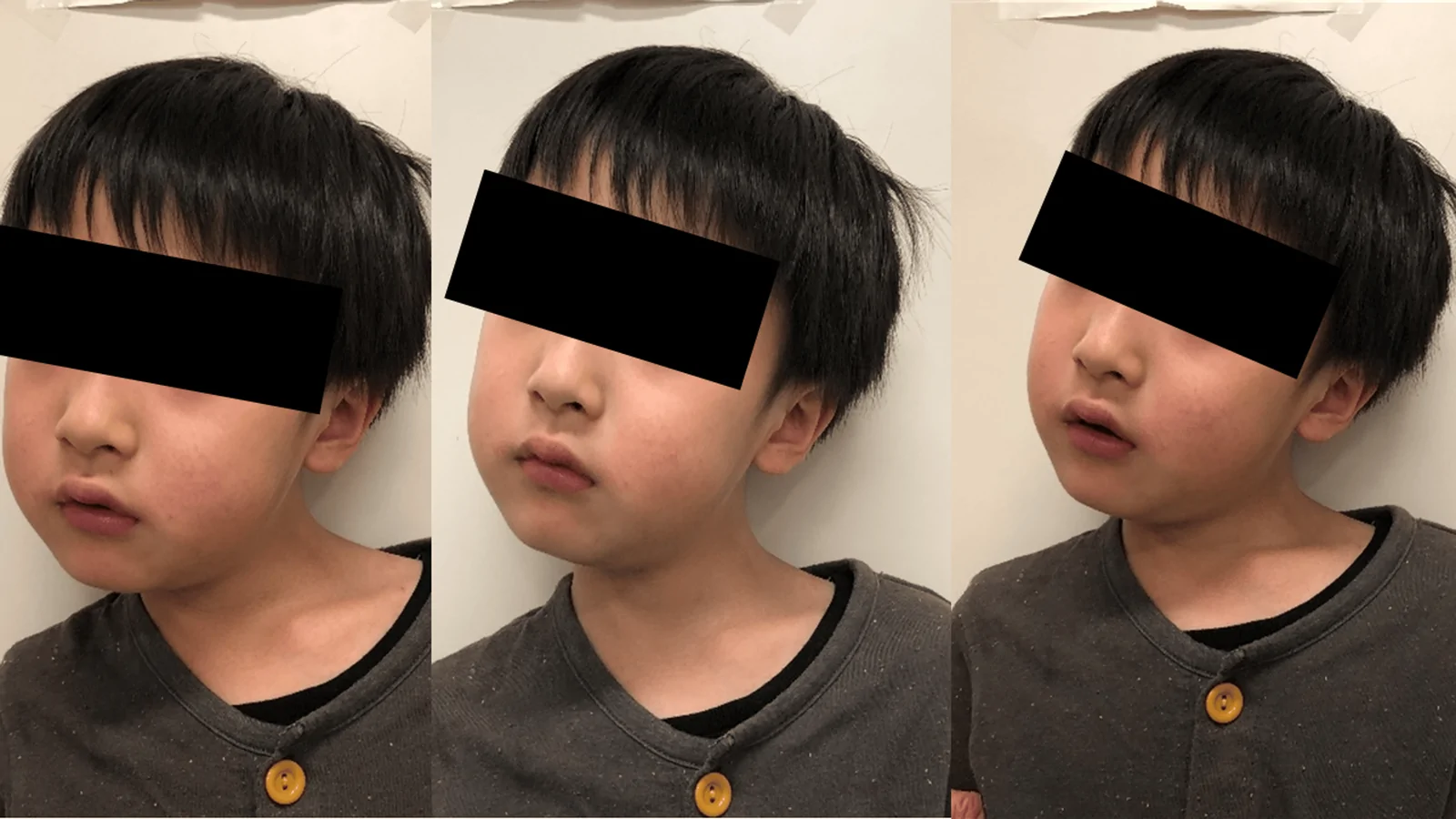
Clinical appearance at the initial visit. Clinical photographs demonstrated pronounced torticollis.

Upon initial presentation at our hospital, marked torticollis was observed (Figure 1).

**Figure 2:**
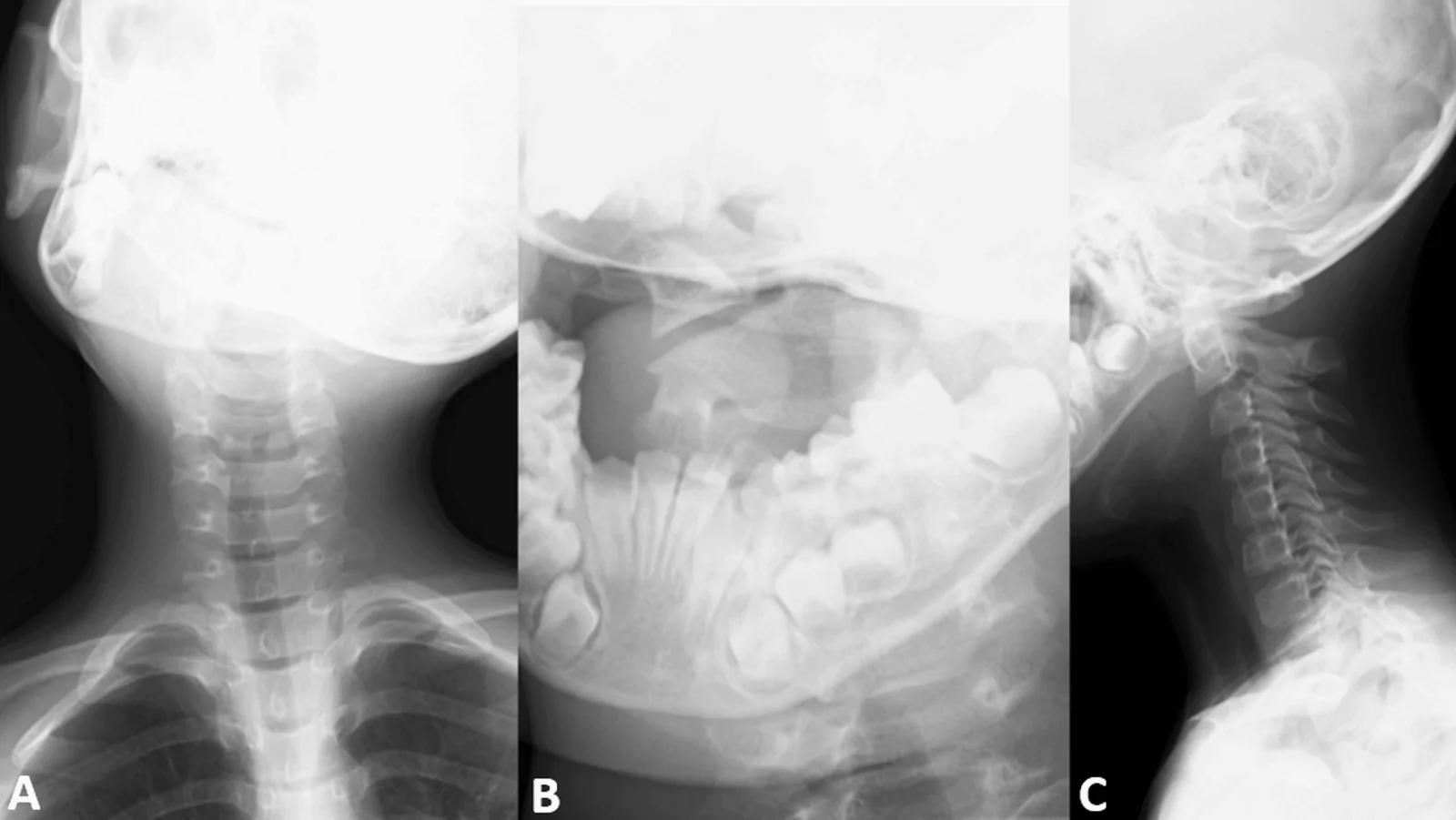
Initial plain radiographs. (A) Anteroposterior view. (B) Open-mouth odontoid view. (C) Lateral view.

The patient reported neck pain but exhibited no motor weakness in either the upper or lower extremities or any sensory disturbance. Gait was preserved, and no spasticity was observed. Laboratory investigations revealed no signs of an inflammatory response. Initial plain radiographs indicated malalignment of the atlantoaxial joint (Figure 2).

**Figure 3:**
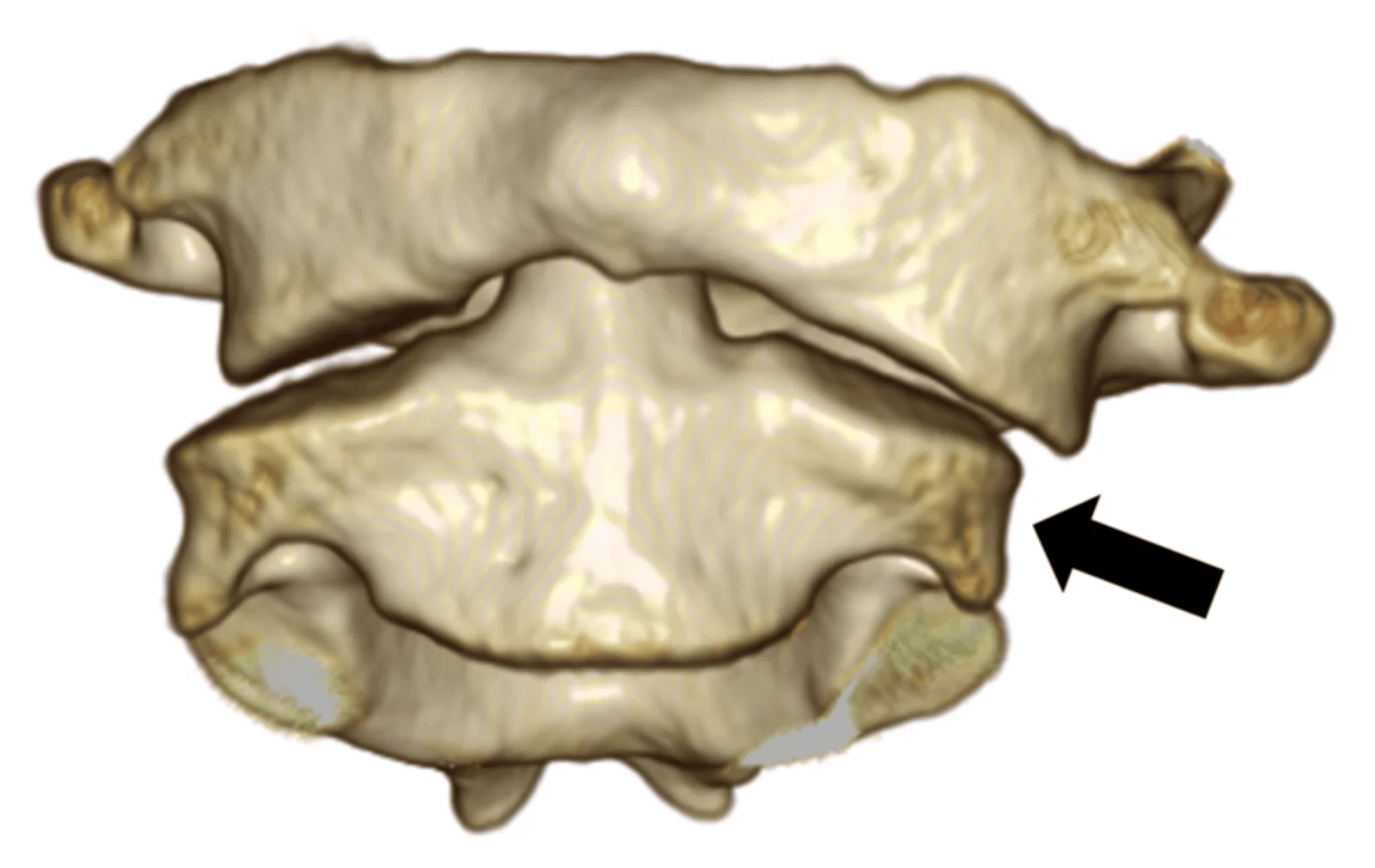
Initial CT findings. Three-dimensional reconstructed CT images obtained at presentation. Despite the two-week interval from symptom onset, mild deformity of the anterior portion of the left C2 facet was already evident. No clival deformity or asymmetry was observed.

Initial CT scans confirmed rotatory subluxation with deformity of the left C2 facet (Figure 3).

Cervical range of motion was assessed, revealing rotation of 40° to the right and 5° to the left. Parameters reported by Yan et al. [2], including the right and left lateral atlanto-dental intervals (LADIs) on coronal CT images, the atlanto-dental interval (AADI) and the retropharyngeal space (RS) on sagittal CT images, and the atlantoaxial rotation angle (ARA) on axial CT images, were measured. The following measurements were observed: right LADI, 1.6 mm; left LADI, 9.0 mm; AADI, 3.6 mm; RS, 9.9 mm; and ARA, 13°. Based on the clinical and CT findings showing rotatory fixation with unilateral facet subluxation and anterior displacement of the atlas, the condition was classified as Fielding type II. MRI was performed to exclude intracranial or intraspinal pathology. Contrast-enhanced MRI was not performed. MRI revealed no spinal cord compression, signs of ligamentous disruption, or inflammatory changes.

**Figure 4:**
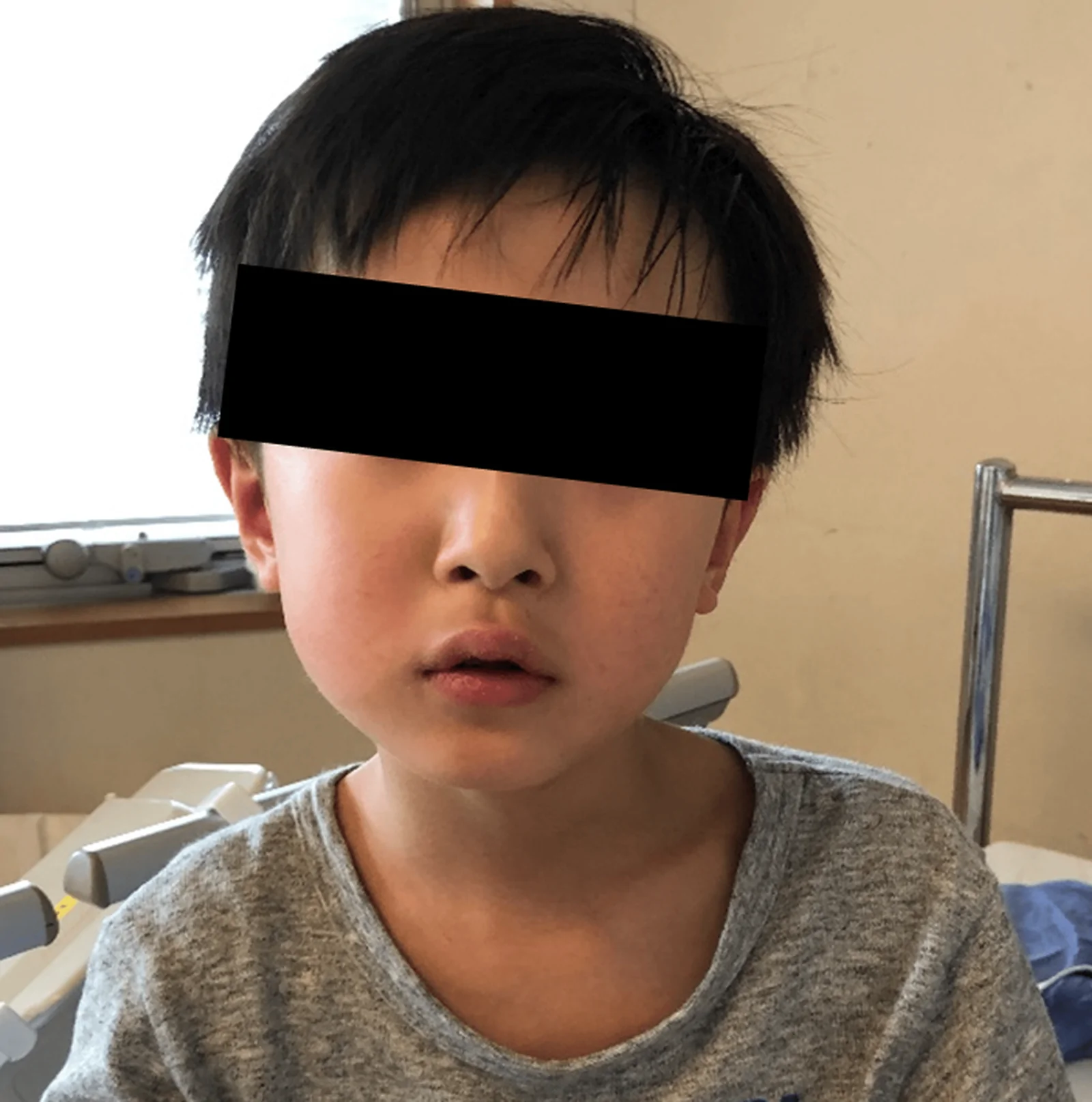
Clinical appearance after three days of traction. Clinical photographs demonstrated partial improvement in torticollis, although residual deformity persisted.

Given the insufficiency of cervical collar immobilization alone, the patient was admitted for traction therapy. After several days of traction, clinical photographs indicated partial improvement of torticollis; however, residual symptoms persisted (Figure 4).

Unfortunately, torticollis recurred shortly after traction cessation. Traction was reinstated multiple times, and although temporary improvements were achieved, symptoms recurred on several occasions after discontinuation (Table 1).

**Table 1:** Clinical course and changes in immobilization during treatment.

Day	Treatment/event	Clinical course
Day 0	Admission; traction initiated	-
Day 10	Switched to a cervical collar	Rotational deformity recurred on the same day, and traction was resumed.
Day 15	Switched to a cervical collar	Neck pain developed the following day, and traction was resumed.
Day 16	Traction resumed	Neck pain worsened the following day.
Day 24	Switched to a cervical collar	-
Day 25	Traction resumed	Torticollis recurred within 1.5 days.
Day 35	Cervicothoracic orthosis applied	-
Day 64	Traction discontinued	-
Day 78	Discharged home	-
Day 238	Switched from the cervicothoracic orthosis to a cervical collar	-
Day 315	Cervical collar discontinued	-

**Figure 5:**
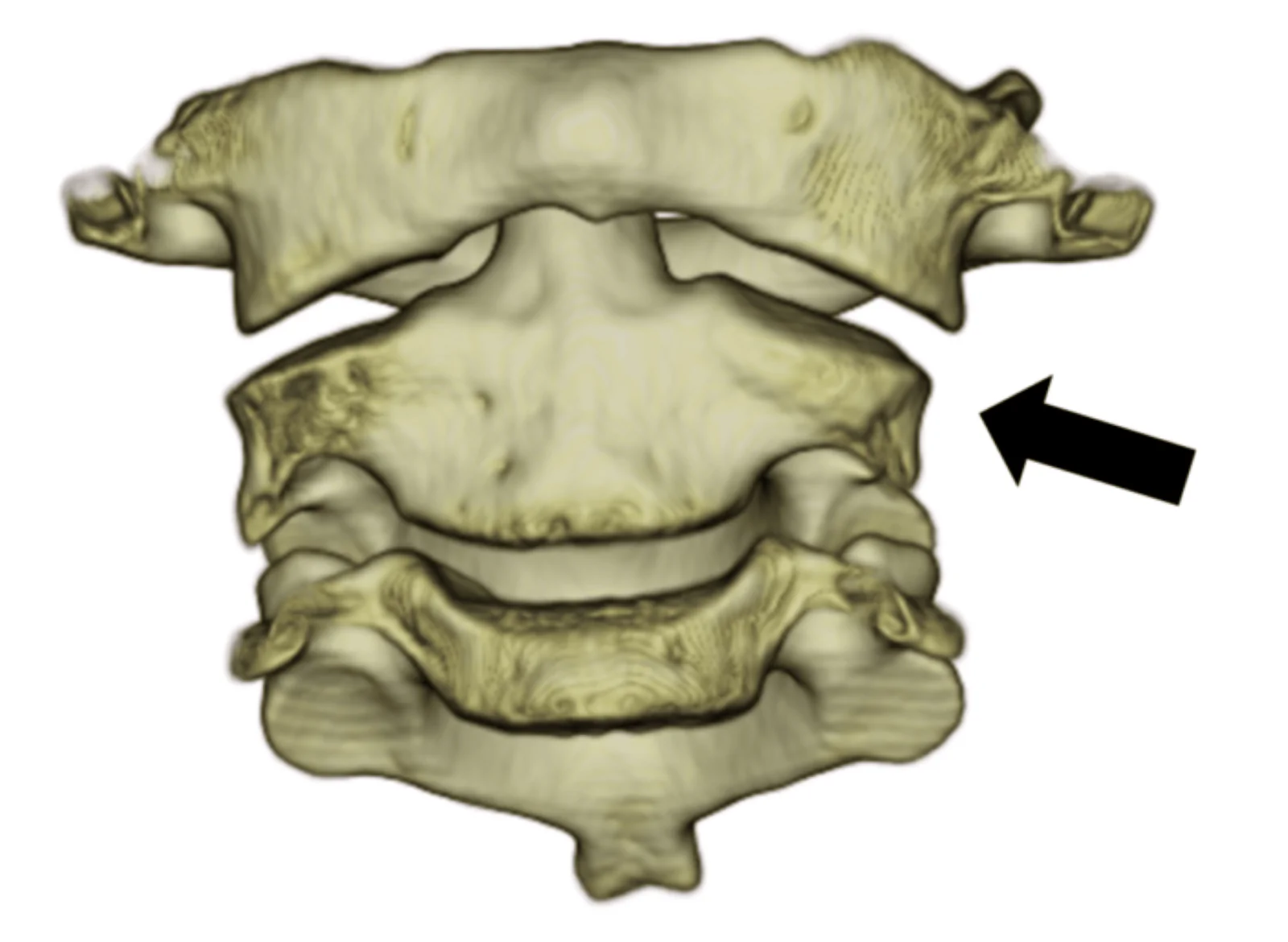
CT findings on hospital day 29. Left C2 facet deformity persisted without evidence of bony union or ankylosis. Clival asymmetry had begun to develop.

**Figure 6:**
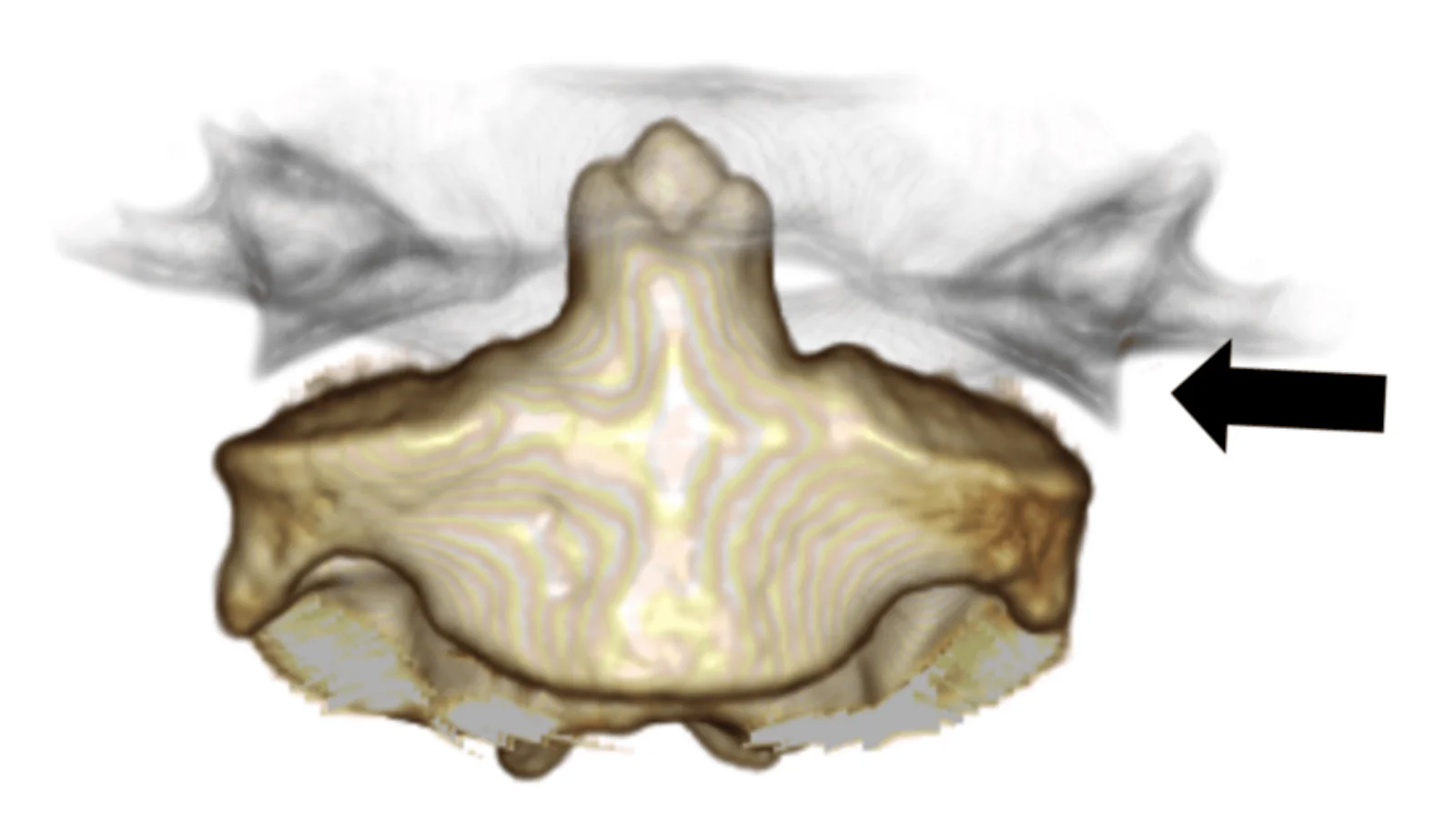
CT findings on hospital day 60. Left C2 facet deformity persisted without evidence of bony union or ankylosis. Clival asymmetry was slightly more pronounced.

CT was repeated on hospital days 29 (Figure 5) and 60 (Figure 6), revealing persistent facet deformity with no radiologic evidence of bony union or ankylotic changes.

Throughout treatment, immobilization was transitioned to a cervicothoracic orthosis (Adfit UD Brace; Advanfit Inc., Yatsushiro, Japan) to enhance rotational control. The patient was instructed to wear the orthosis continuously. Outpatient follow-up examinations indicated satisfactory compliance, and neutral alignment was maintained throughout the treatment period.

**Figure 7:**
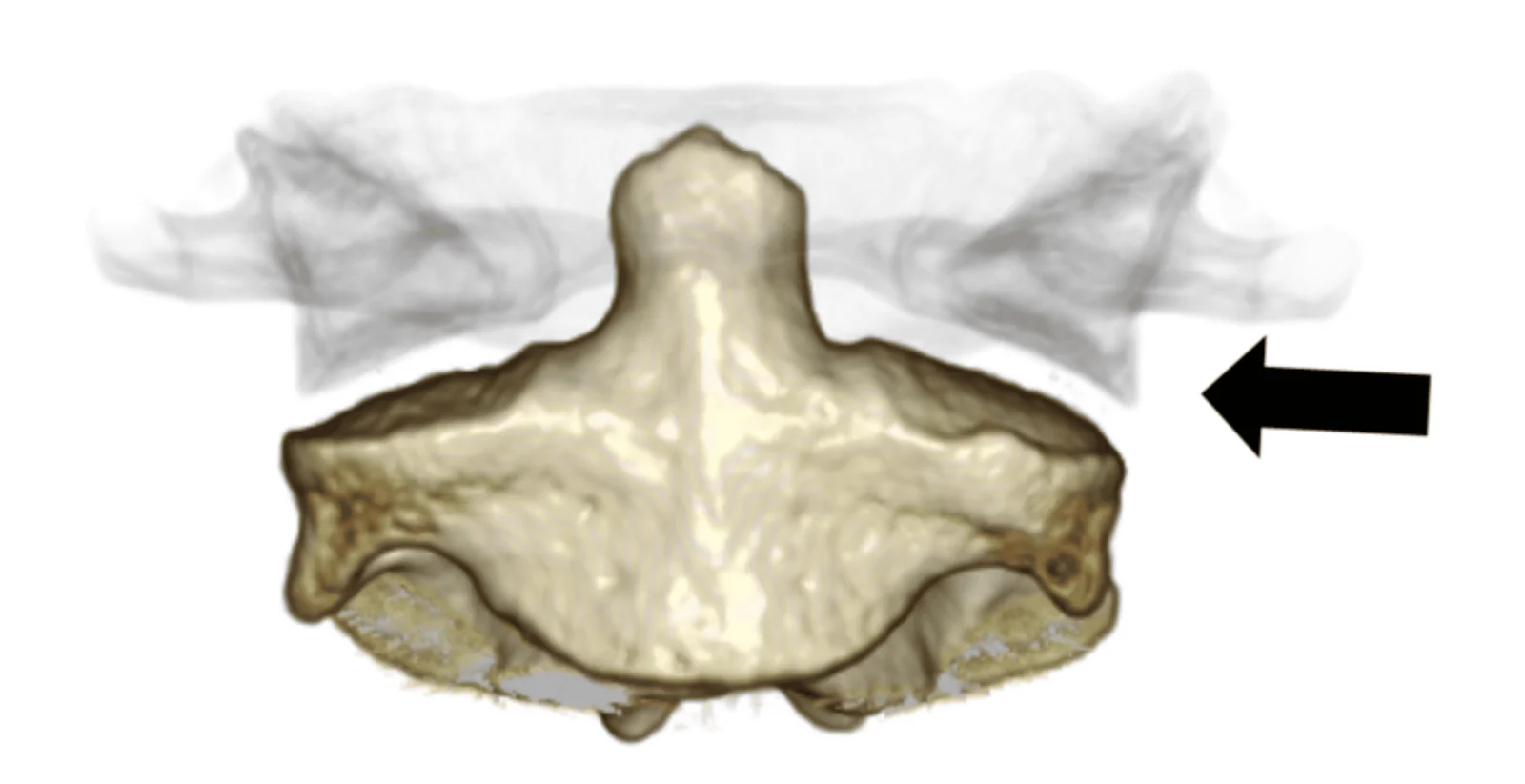
CT findings six months after onset. Residual deformity of the left C2 facet persisted; however, clival asymmetry had nearly resolved, and there was no evidence of bony fusion or ankylosis.

After a successful trial home leave, the patient was discharged home. He was permitted to return to school. The patient wore the cervicothoracic orthosis throughout the day, except while bathing and sleeping. At the six-month follow-up, CT demonstrated mild residual facet deformity (Figure 7); however, neutral cervical alignment was maintained. He was subsequently permitted to resume sports activities.

**Figure 8:**
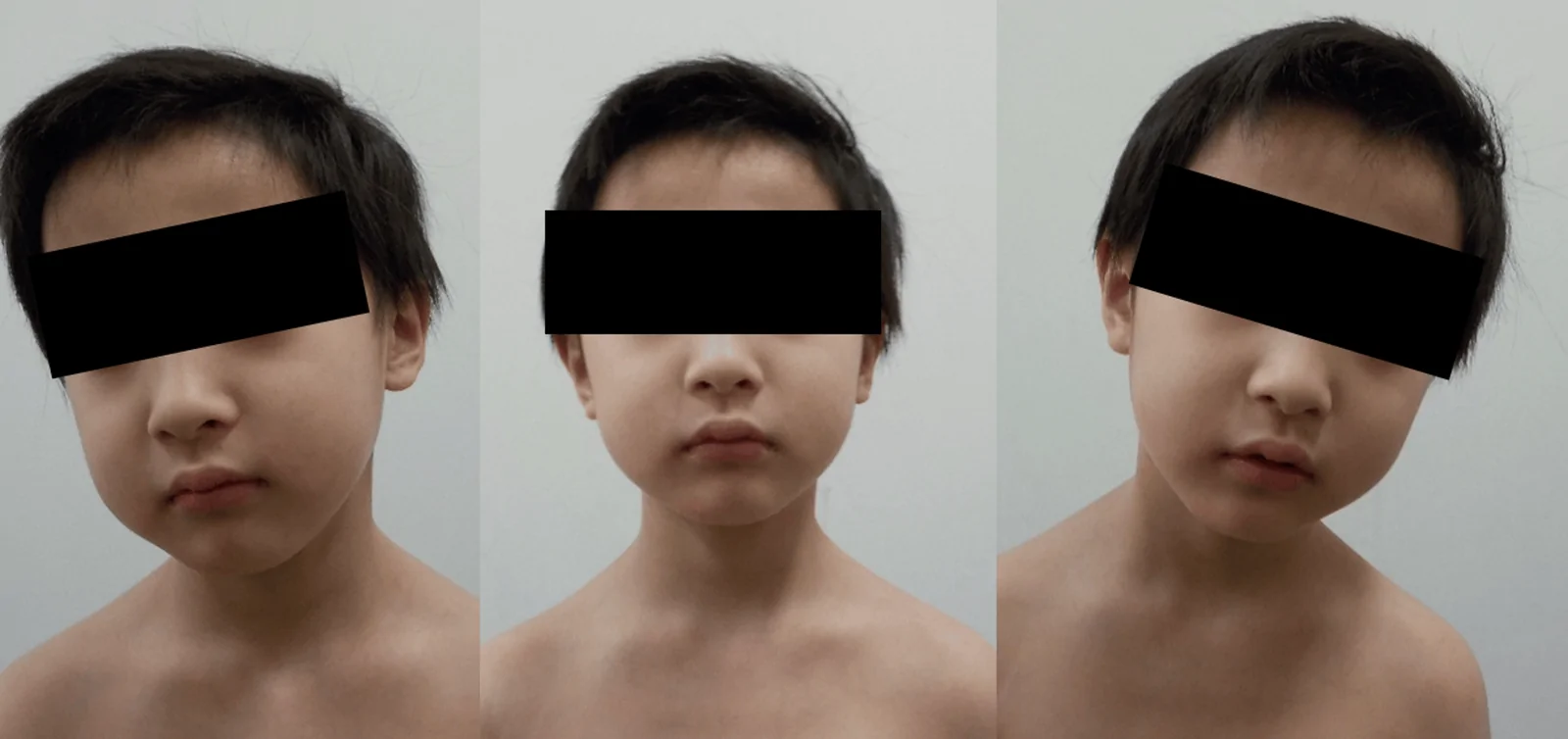
Clinical appearance six months after onset. Clinical photographs demonstrated neutral cervical alignment and marked improvement in torticollis.

However, cervical range of motion was unrestricted (Figure 8), and the patient was free of neck pain, allowing discontinuation of orthotic immobilization.

**Figure 9:**
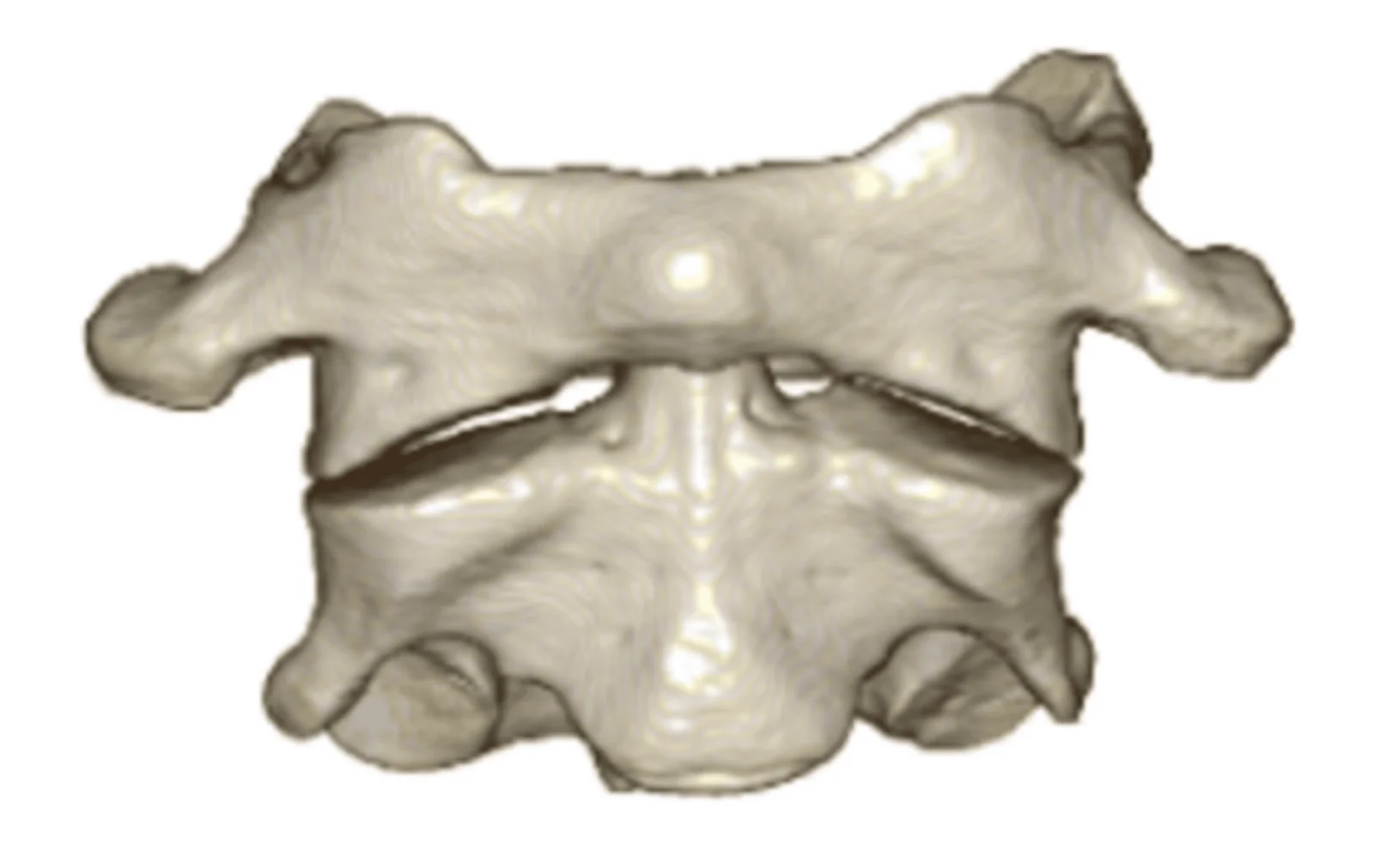
CT findings at the five-year follow-up. Clival deformity had resolved. Slight residual deformity of the left C2 facet persisted; however, there was no evidence of bony ankylosis or degenerative changes.

At the five-year follow-up, the patient’s height was 171 cm, and his weight was 63.0 kg. CT images revealed only slight residual facet deformity without evidence of bony ankylosis or degenerative arthritic changes (Figure 9).

**Figure 10:**
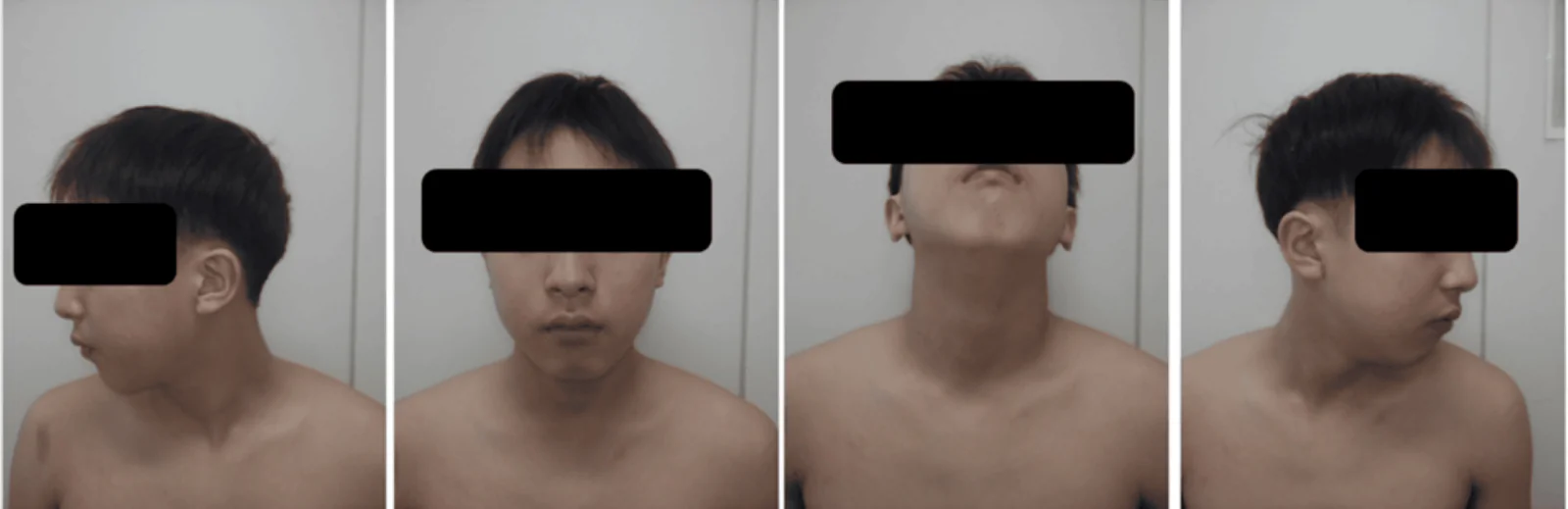
Clinical appearance at the five-year follow-up. The patient reported no neck pain. Clinical photographs demonstrated full and symmetric cervical range of motion.

Clinical photographs at the five-year follow-up demonstrated the absence of pain and full, symmetric cervical range of motion, including bilateral rotation (Figure 10).

At the latest follow-up, the patient enjoyed unrestricted school life with no recurrent episodes of torticollis, and no neurological sequelae were observed. Although the patient was clinically asymptomatic, follow-up CT was performed at six months and five years because osteophyte formation and facet deformity had already been present at the initial examination. The purpose was to evaluate for recurrent subluxation, progression of the residual deformity, and possible late bony changes during growth. Because osteophyte formation and facet deformity were already present at the initial visit, long-term follow-up was considered necessary to monitor for recurrence, residual deformity, and potential late changes associated with growth. The patient was therefore followed for five years. At the five-year follow-up, he reported no neck pain or limitations in activities of daily living.

## Discussion

The present case demonstrates that prolonged AARF may be successfully managed with a remodeling-based strategy without manipulation under general anesthesia or halo vest immobilization when neutral alignment can be maintained with a cervicothoracic orthosis. Previous reports of remodeling therapy used closed reduction under general anesthesia followed by halo vest immobilization [5-7]. In contrast, our patient was young, had substantial remodeling potential, and maintained neutral alignment with a cervicothoracic orthosis.

In children, torticollis is most frequently caused by benign conditions such as muscle spasm, infection, trauma, or AARF. However, several studies underscore that a small yet clinically significant proportion of pediatric torticollis cases can be attributed to intracranial or intraspinal pathology. In a large emergency department cohort of 1,409 children, Raucci et al. [4] reported that oncologic etiologies, including posterior fossa and brainstem tumors, accounted for approximately 1.2% of all torticollis presentations. This finding highlights the importance of remaining vigilant for CNS disease, particularly when torticollis is atypical or fails to improve with initial management.

Although AARF usually improves with conservative treatment, the present patient had persistent symptoms and already showed facet deformity and bony changes at the initial visit. Previous reports have shown that torticollis may occasionally be the first or only manifestation of posterior fossa or cervical spinal cord tumors [8,9]. Therefore, MRI imaging was performed to exclude intracranial and intraspinal pathology before continuing treatment for AARF.

In the present case, brain and cervical spine MRI were performed because symptoms persisted beyond the typical clinical course. No evidence of intracranial or intraspinal pathology was identified, allowing the patient to be managed as having refractory AARF.

Clinically, AARF typically resolves within several days, and prolonged treatment courses, such as that observed in the present case, are uncommon. Consequently, there is currently no widely accepted consensus regarding the management of prolonged or refractory cases. Several case reports have described various treatment strategies for chronic AARF [5-7]. Notably, Ishii et al. described a remodeling-based strategy aimed at preventing recurrent subluxation by addressing facet deformity [7].

In our patient, torticollis repeatedly recurred after Glisson traction was discontinued, despite temporary improvement in alignment. A CT scan obtained shortly after the onset of torticollis had already indicated mild deformity of the left C2 facet, suggesting that structural changes might have been present at an early stage; however, the etiology of this deformity remains unclear. The patient had no prior history of torticollis, no palpable mass of the sternocleidomastoid muscle, and maintained a neutral head position before symptom onset. Furthermore, conventional traction alone was insufficient to prevent recurrence. These observations indicate that the present case did not follow the typical clinical trajectory of acute AARF, and the mechanism underlying the early facet deformity remains undetermined.

Regarding treatment selection, manipulation under general anesthesia and halo vest fixation were not pursued because neutral alignment could be manually achieved and maintained with external immobilization. In addition, halo vest immobilization necessitates invasive pin insertion, which the patient’s family preferred to avoid. Operative stabilization was considered a backup option if neutral alignment could not be achieved or sustained. Given that a neutral position could be maintained using a cervicothoracic orthosis, outpatient management with continuous orthosis wear was adopted as a conservative approach. At six months after symptom onset, torticollis had resolved, cervical range of motion was symmetric, and the patient remained symptom-free in daily activities. Long-term follow-up CT at five years revealed only minimal residual C2 facet deformity without progression of degenerative changes or bony ankylosis.

In this patient, treatment decisions were based on symptom duration, clinical severity, and the ability to achieve and maintain neutral cervical alignment. When neutral alignment can be achieved with traction but recurrence occurs after traction is discontinued, immobilization with a cervicothoracic orthosis may represent an additional conservative option in carefully selected patients before more invasive treatment is considered.

This case also emphasizes the importance of considering CNS imaging in children with persistent or atypical torticollis, even when AARF is clinically suspected. Early exclusion of intracranial pathology is particularly crucial when the clinical course deviates from the expected rapid improvement observed in most AARF cases.

Limitations

This report documents a single case; therefore, the generalizability of our findings is limited. In addition, the multiple treatment modalities employed, including traction and orthotic immobilization, preclude determination of each intervention’s relative contribution.

## Conclusions

We successfully treated a pediatric patient with AARF accompanied by early facet deformity using remodeling therapy without general anesthesia or halo vest immobilization. Sustained neutral alignment may facilitate remodeling and prevent the progression of facet deformity. The patient showed favorable long-term clinical and radiographic outcomes, including resolution of neck pain and torticollis, good cervical range of motion at the final follow-up, CT-confirmed facet remodeling, and no recurrence during five years of follow-up. This case provides valuable clinical insights into the management of refractory AARF.
